# Current concepts and dilemmas in idiopathic interstitial pneumonias

**DOI:** 10.12688/f1000research.9601.1

**Published:** 2016-11-10

**Authors:** Jay H. Ryu, Teng Moua, Natalya Azadeh, Misbah Baqir, Eunhee S. Yi

**Affiliations:** 1 Division of Pulmonary and Critical Care Medicine, Mayo Clinic, Rochester, MN, USA; 2 Division of Anatomic Pathology, Mayo Clinic, Rochester, MN, USA

**Keywords:** idiopathic interstitial pneumonia, lung disease, parenchymal

## Abstract

Idiopathic interstitial pneumonias comprise approximately one-third of interstitial lung diseases (also called diffuse parenchymal infiltrative lung diseases). The classification of idiopathic interstitial pneumonias has undergone several revisions since the initial description of 40 years ago, and the most recent version was published in 2013. Although some aspects have been clarified, this group of heterogeneous disorders continues to be a source of confusion and misunderstanding in clinical applications. In this article, we explore several topical themes in the evaluation and management of patients with idiopathic interstitial pneumonias.

## Introduction

Idiopathic interstitial pneumonias (IIPs) comprise approximately one-third of interstitial lung diseases (ILDs) but remain a difficult concept to grasp for clinicians and scientists. These disorders represent a heterogeneous group of diffuse parenchymal lung diseases that are defined on the basis of histopathologic pattern combined with clinical and radiologic features. There continue to be misunderstanding and misapplication of concepts pertaining to IIPs, which may lead to suboptimal management and patient outcomes. Revisions in definitions and classification of IIPs have likely contributed to this problem. Some patients with IIPs remain unclassifiable owing to discordant or overlapping clinical, imaging, and histopathologic features or lack of inter-observer agreement ^1, 2^. In this article, we will explore the current concepts and dilemmas regarding this group of diffuse parenchymal lung diseases.

## Historical background

Averill A. Liebow, a pathologist, provided the initial definition and classification of interstitial pneumonias in 1974 ^3^. He described “interstitial pneumonia” as “a type of response to injury in the lung that takes place predominantly in the supporting structures rather than within the alveoli” ^3^. He noted that although interstitial fibrosis may be a possible consequence of this injury, there is diversity in the initial morphology of these disorders and associated clinical correlates. This early concept classified interstitial pneumonias based on morphologic (histologic) criteria into five types: “usual” interstitial pneumonia (most commonly encountered), bronchiolitis obliterans with classical interstitial pneumonia, desquamative interstitial pneumonia, lymphoid interstitial pneumonia, and giant cell interstitial pneumonia.

Over the subsequent 40 years, the classification of interstitial pneumonias has undergone several revisions ^4– 6^. Similarly, the concept of idiopathic pulmonary fibrosis (IPF) has also been modified; the initial definition of IPF (also called cryptogenic fibrosing alveolitis) included several distinct types of interstitial pneumonias as well as those with identifiable underlying diseases (i.e. not idiopathic), e.g. connective tissue diseases ^7, 8^. Thus, the definition of IPF became narrowed to that of usual interstitial pneumonia of unknown cause, i.e. no identifiable cause or underlying disease responsible for the fibrotic lung disease. During the process of these conceptual changes, the term “idiopathic” became attached to the classification of interstitial pneumonias, i.e. IIPs ^4– 6^. The most recent version of the IIPs lists three major categories: major, rare, and unclassifiable. The first group includes IPF, idiopathic nonspecific interstitial pneumonia, respiratory bronchiolitis–ILD, desquamative interstitial pneumonia, cryptogenic organizing pneumonia, and acute interstitial pneumonia. The second comprises idiopathic lymphoid interstitial pneumonia and idiopathic pleuroparenchymal fibroelastosis. IPF, i.e. usual interstitial pneumonia of unidentifiable cause, remains the most commonly encountered form of IIP ^8, 9^.

## Are idiopathic interstitial pneumonias truly idiopathic?

Disease classifications provide a framework for conceptualization and communication but inevitably change over time as additional insight and knowledge are gained. Although the current IIP classification inserts the term “idiopathic” for several types of interstitial pneumonias in an attempt to distinguish IIP from interstitial pneumonia with identifiable causes or underlying disease, several dilemmas remain.

Most experts acknowledge that respiratory bronchiolitis-associated ILD and desquamative interstitial pneumonia in the majority of patients represent smoking-related lung diseases ^10– 13^. Indeed, smoking has been associated with an expanding spectrum of ILDs including pulmonary Langerhans cell histiocytosis, acute eosinophilic pneumonia, combined pulmonary fibrosis and emphysema syndrome, and smoking-related interstitial fibrosis. The main mode of treatment for subjects with smoking-related ILDs is smoking cessation rather than the use of immunosuppressive or antifibrotic agents employed in other forms of IIPs. Thus, continued inclusion of respiratory bronchiolitis-associated ILD and desquamative interstitial pneumonia under the umbrella of IIPs seems difficult to justify other than for historical reasons.

Nonspecific interstitial pneumonia refers to a pattern of lung injury characterized by chronic interstitial inflammation and fibrosis that lacks the typical features of usual interstitial pneumonia such as temporal heterogeneity with fibroblast foci and honeycomb fibrosis ^4, 5, 14^. Nonspecific interstitial pneumonia is known to be associated with connective tissue diseases such as scleroderma and polymyositis. In recent years, it has become apparent that many patients with “idiopathic” nonspecific interstitial pneumonia develop features of definable connective tissue diseases such as inflammatory arthritis, rash, and sclerodactyly. Demographic features such as female gender and younger age at presentation support a greater likelihood of underlying connective tissue disease. In this regard, a recently proposed concept of “interstitial pneumonia with autoimmune features” (IPAF) is relevant ^15^. This term recognizes the observation that many patients with IIPs, particularly nonspecific interstitial pneumonia, have features suggestive of an underlying autoimmune process but do not meet criteria for definable connective tissue disease and attempts to bring together what has previously been called “undifferentiated connective tissue disease-associated ILD” and “lung-dominant connective tissue disease” ^15^. The proposed criteria for the diagnosis of IPAF include evidence of an interstitial pneumonia (radiologic or morphologic) of unknown cause and at least one feature from two of three domains: clinical domain (clinical features suggestive of autoimmune disease), serologic domain (circulating autoantibodies), and morphologic domain (high-resolution computed tomography [HRCT] or histopathologic features). It is hoped that this consensus in nomenclature and classification criteria will bring about a more informed path to gaining insight into this group of patients. However, some patients with nonspecific interstitial pneumonia will not have an identifiable cause or underlying disease despite a comprehensive evaluation, i.e. remain idiopathic.

Other forms of IIPs such as organizing pneumonia and lymphocytic interstitial pneumonia are rarely idiopathic. Unfortunately, this is not widely appreciated by clinicians, who may neglect to reassess such patients for identifiable causes and underlying diseases. Furthermore, although patients with usual interstitial pneumonia commonly have IPF, other diagnostic possibilities remain, even for those with a surgical lung biopsy confirmation. Several studies have demonstrated that the histologic pattern of usual interstitial pneumonia can be seen in patients with chronic hypersensitivity pneumonitis as well as connective tissue diseases ^16– 19^. Thus, we wonder whether the continued use of the original term “interstitial pneumonias” coined by Liebow may have prevented some of the confusion associated with the current term “IIPs” in referring to this group of ILDs.

## What is the role of lung biopsy?

As discussed, the initial concept and classification of interstitial pneumonias were defined by histopathologic features. The advent of HRCT has allowed noninvasive diagnosis of IIPs to be possible in many cases. Decreasing need for lung biopsy, particularly surgical lung biopsy, has evolved through a better understanding of radiologic correlates of interstitial pneumonias. For example, a HRCT pattern characterized by lower lobe and peripherally predominant reticular and honeycomb changes in the absence of other features such as micronodules, cysts, or ground glass is highly predictive of usual interstitial pneumonia and does not require biopsy confirmation of the underlying histopathologic pattern ^8^. However, the absence of typical HRCT features such as peripheral honeycombing does not exclude the possibility of usual interstitial pneumonia ^20^. Respiratory bronchiolitis-associated ILD can generally be diagnosed without a biopsy in smokers who manifest patchy ground-glass opacities and centrilobular nodules ^6^.

The “definitive” method of obtaining tissue in patients with suspected ILD is surgical lung biopsy, usually performed as a video-assisted thoracoscopic procedure. Surgical lung biopsy clearly has a role in defining the underlying histopathologic pattern in patients with nondiagnostic HRCT findings. For example, nonspecific interstitial pneumonia, desquamative interstitial pneumonia, and hypersensitivity pneumonia may all be characterized by diffuse ground-glass opacities in the lung but entail different treatment strategies. However, surgical lung biopsy is associated with significant risks, particularly for those patients with advanced age, comorbid conditions, IPF, and connective tissue-related ILDs ^21– 23^. Thus, the benefits and risks of a surgical lung biopsy need to be individualized according to the clinical context with consideration of diagnostic possibilities, anticipated biopsy results, and potential implications on management decisions.

Bronchoscopic biopsy usually performed with forceps is generally not diagnostic in patients with IIPs. However, the recent advent of cryobiopsy may increase the role of bronchoscopic biopsy in this clinical setting. Several studies have demonstrated increased diagnostic rate with bronchoscopic cryobiopsy even for patients with suspected IPF ^24– 27^. For example, Tomassetti and colleagues ^24^ recently demonstrated bronchoscopic cryobiopsy findings to have an impact similar to surgical lung biopsy in a multidisciplinary diagnostic process for patients with ILDs. Although bronchoscopic cryobiopsy yields larger specimens with fewer crush artifacts, the optimal technique remains to be determined since cryobiopsy may be associated with increased rates of bleeding and pneumothorax.

## Multidisciplinary discussion or clinical reasoning?

Multidisciplinary discussion (MDD) has been touted as the “gold standard” process by which a diagnosis is achieved in cases of IIPs ^6, 8, 28, 29^. This conclusion has been primarily based on the observation of improved inter-observer agreement and diagnostic confidence resulting from dynamic interactions among clinicians, radiologists, and pathologists. However, the costs of multidisciplinary team meetings are high and the impact on clinical outcomes is unclear ^30– 32^. MDD for the diagnosis of IIPs is likely not a realistic option outside of academic medical centers.

The emphasis on the role of MDD in the diagnosis of IIPs has come about, in part, because of unrealistic expectations of clinicians as to the role of histopathologic patterns in the diagnosis of IIPs. Histopathologic patterns have varying morphologic specificities in identifying diseases and associated etiologies. Histologic pattern should not be confused with a diagnosis and the importance of clinical evaluation underestimated. For example, organizing pneumonia pattern is very nonspecific and can be seen in many diseases including infections, connective tissue disease-related ILD, aspiration, drug-induced lung injury, and many others. Thus, the diagnoses of IIPs, as well as ILDs in general, require judicious integration of relevant clinical, radiologic, and histopathologic (when available) data, i.e. clinical reasoning ^33– 35^. There are situations where a face-to-face interaction or verbal communication among the clinician, radiologist, and pathologist can be invaluable, but it is unrealistic to expect MDDs to occur routinely in the diagnostic evaluation of patients with suspected IIPs in most clinical practice settings. Ultimately, it is the essential role of the clinician to identify and integrate pivotal information derived from relevant disciplines in defining a diagnosis and implications thereof for the individual patient. Those patients with problematic and unresolved issues can be referred to an ILD center with appropriate expertise, MDD capability, and access to relevant clinical trials.

## What is the cause of acute exacerbations?

“Acute exacerbation” is a term that has been used to describe acute unexplained deterioration in respiratory status occurring during the clinical course of patients with IPF ^36^. Over the past several years, this phenomenon has been recognized to occur in patients with other forms of fibrotic lung diseases, including chronic hypersensitivity pneumonitis, desquamative interstitial pneumonia, and connective tissue-related ILD ^37, 38^. Although the initial consensus definition of acute exacerbation required the exclusion of infections, pulmonary embolism, and other identifiable etiologies, a recent report from an international working group suggests that the definition be broadened to include any respiratory event characterized by new bilateral infiltrates not explained by heart failure or fluid overload ^39^. This revised definition for acute exacerbation is meant to be more inclusive and more applicable in clinical practice.

Acute exacerbation occurring in patients with ILDs is associated with an alarmingly high short-term mortality rate of at least 50% ^36, 40^. Histopathologic pattern associated with this acute lung injury is diffuse alveolar damage in most cases, sometimes organizing pneumonia ^36, 40– 42^. However, the underlying cause for this phenomenon remains unclear as to whether it represents an accelerated phase of the progressive fibrotic lung process or superimposed injury induced by an external trigger such as viral infection, microaspiration, or some other form of inhalational injury. Although systemic glucocorticoid therapy is commonly used in the treatment of acute exacerbation in these patients, there is no clear evidence that such a strategy or other forms of treatment are effective.

## Conclusions

Although recent revisions in the concept of IIPs have clarified some issues, the current classification scheme of IIPs can be confusing for clinicians as well as radiologists and pathologists. Some forms of IIPs included in the current classification are not idiopathic, e.g. respiratory bronchiolitis-associated ILD and desquamative interstitial pneumonia, and some patients with IIPs may eventually manifest signs of a definable connective tissue disease. The role and the risks of surgical lung biopsy in the diagnosis of IIPs are becoming better defined. Although identification of the underlying histopathologic pattern can be important, it should be clinical reasoning that integrates all relevant data which is crucial in achieving a diagnosis. It is hoped that the recently revised definition of acute exacerbation will lead to a better understanding and ultimately management of this deadly phenomenon.

## Abbreviations

HRCT, high-resolution computed tomography; IIP, idiopathic interstitial pneumonia; ILD, interstitial lung disease; IPAF, interstitial pneumonia with autoimmune features; IPF, idiopathic pulmonary fibrosis.
